# Exoskeleton-based exercises for overground gait and balance rehabilitation in spinal cord injury: a systematic review of dose and dosage parameters

**DOI:** 10.1186/s12984-024-01365-2

**Published:** 2024-05-05

**Authors:** Patrik Nepomuceno, Wagner H. Souza, Maureen Pakosh, Kristin E. Musselman, B. Catharine Craven

**Affiliations:** 1 KITE Research Institute, University Health Network, Toronto, ON Canada; 2 Graduate Program in Health Promotion, Department of Health Sciences, University of Santa Cruz do Sul, Santa Cruz do Sul, RS Brazil; 3 Department of Physical Therapy, Temerty Faculty of Medicine, University of Toronto, Toronto, ON Canada; 4 Department of Medicine, Temerty Faculty of Medicine, University of Toronto, Toronto, ON Canada; 5 Institute of Health Policy Management and Evaluation, University of Toronto, Toronto, Canada; 6 Rehabilitation Sciences Institute, Temerty Faculty of Medicine, University of Toronto, Toronto, Canada

**Keywords:** Exoskeleton, Gait, Neurorehabilitation, Overground training, Posture, Spinal cord injury

## Abstract

**Background:**

Exoskeletons are increasingly applied during overground gait and balance rehabilitation following neurological impairment, although optimal parameters for specific indications are yet to be established.

**Objective:**

This systematic review aimed to identify dose and dosage of exoskeleton-based therapy protocols for overground locomotor training in spinal cord injury/disease.

**Methods:**

A systematic review was conducted in accordance with the Preferred Reporting Items Systematic Reviews and Meta-Analyses guidelines. A literature search was performed using the CINAHL Complete, Embase, Emcare Nursing, Medline ALL, and Web of Science databases. Studies in adults with subacute and/or chronic spinal cord injury/disease were included if they reported (1) dose (e.g., single session duration and total number of sessions) and dosage (e.g., frequency of sessions/week and total duration of intervention) parameters, and (2) at least one gait and/or balance outcome measure.

**Results:**

Of 2,108 studies identified, after removing duplicates and filtering for inclusion, 19 were selected and dose, dosage and efficacy were abstracted. Data revealed a great heterogeneity in dose, dosage, and indications, with overall recommendation of 60-min sessions delivered 3 times a week, for 9 weeks in 27 sessions. Specific protocols were also identified for functional restoration (60-min, 3 times a week, for 8 weeks/24 sessions) and cardiorespiratory rehabilitation (60-min, 3 times a week, for 12 weeks/36 sessions).

**Conclusion:**

This review provides evidence-based best practice recommendations for overground exoskeleton training among individuals with spinal cord injury/disease based on individual therapeutic goals – functional restoration or cardiorespiratory rehabilitation. There is a need for structured exoskeleton clinical translation studies based on standardized methods and common therapeutic outcomes.

**Supplementary Information:**

The online version contains supplementary material available at 10.1186/s12984-024-01365-2.

## Introduction

Over the past decade, lower limb robotic technologies have been increasingly applied in neurorehabilitation [1, 2]. Essentially anthropomorphic in concept, these powered mechanical devices are used for locomotor training [3] and are classified as end-effectors or exoskeletons [4]. The first one generates movements from the distal segment through a haptic interface [5], while the latter encompass independent robot joints guided in a pre-programmed trajectory which is further classified as unilateral or bilateral [4]. Among such technologies, exoskeletons are reportedly useful to promote mobility in individuals with locomotor dysfunction, including those with complete lower extremity paralysis [6]. Exoskeletons often do so through motorized actuators that assist hip, knee, and ankle motion in dynamic orthoses capable of supporting, stabilizing and reciprocally progressing the lower limbs [4]. Newer generation devices offer training modes which allow therapists to manually trigger and control steps, in addition to adaptive and variable assistive features for individuals with incomplete injuries and a fair prognosis for voluntary active movement and functional recovery.

More recently, an alternative robotic exoskeleton classification was suggested based on four categories: end-effectors (e.g., Haptic Walker), grounded exoskeletons (e.g., Lokomat), wearable exoskeletons (e.g., Ekso and ReWalk) and soft exoskeletons (e.g., Myosuit) [7]. These devices seem especially promising as strategies to improve balance and walking abilities [8, 9], two of the most frequent goals following subacute or chronic spinal cord injury/disease (SCI/D) [7, 10]. The first, characterized by physiologic responses at a cellular level (e.g., glial scars), occurs within a few weeks after the injury [11–13]. Conversely, the latter is achieved as of 6 months after the injury. In traumatic SCI, the interval between the acute (< 30 days) and chronic (> 6 months) phases has been labelled the intermediate phase [14].

In terms of motor support, exoskeletons offer different types of assistance including active (equipment performs the movement, partially or totally, through powered assistance to the user); passive (device does not offer powered assistance to the movement, users execute by themselves); active-assisted (offers powered assistance to complete movements initiated by the user); resistive (offers resistance to movements initiated by the user); and interactive (uses feedback to correct movements based on interactions between actuators and control strategies) [4, 7, 15]. Understanding these different levels of assistance is important to account for the variable forms of haptic feedback involved in robotic motor training which can either enhance or degrade motor performance depending on the patient’s impairments and abilities (e.g., novice learners vs. advanced learners, subacute vs. chronic patients, those with autonomic or sensory function, presence or absence of spasticity, etc.) [16]. Prior publications with these variable assistive devices have shown that gait and balance training with exoskeletons contribute to increased energy expenditure, muscle activation/recruitment and weight bearing [17–20], in addition to improved independence and health-related quality of life [21]. These outcomes are often achieved in response to neurorecovery fostered by functional restoration programs[22]. *Functional Restoration interventions* focus on the refinement of sensorimotor function in daily living. That ability is associated with the stimulation of remaining neural connections that even in SCI/D re-enable sensorimotor function following repeated exposure to directed stimuli, hence yielding [23] greater motor and autonomic recovery [23, 24].

Specific to SCI/D, a recent study of exoskeleton-based rehabilitation among individuals with subacute injury reported that exposure to sixteen 30-min sessions of robotic-assisted gait training led to a significant improvement in gait as measured by the Walking Index SCI II (WISCI-II), which translates to more functional gait and activities of daily living [25]. Moreover, Tamburella et al. [26] reported that individuals living with SCI/D could walk significantly faster, with longer steps and reduced gait cycles after rehabilitation with a powered exoskeleton. Similarly, Okawara et al. [27] reported gains in the 10-Meter Walk Test (10MWT), Time Up and Go (TUG) and Berg Balance Scale (BBS) after twenty 60-min sessions of body weight supported treadmill training (BWSTT) with a hybrid-assisted limb system. These results, however, were only observed in SCI/D patients with prior high walking ability as measured by the WISCI-II. In a similar population, Baunsgaard et al. [28] performed twenty-four 60-min sessions of robotic exoskeleton gait training, which resulted in improvements in the 10MWT, TUG and BBS, however with no treadmill or body weight support. The aforementioned results suggest that individuals living with subacute spinal cord lesions (< 1 year) are most likely to experience therapeutic benefits. However, individuals living with chronic SCI/D may also benefit from these interventions. While neuroplasticity is primarily expected at earlier phases after SCI/D, improvements are still attainable at later stages, specifically in response to coordinated, repeated motor stimuli as fostered by exoskeletons [4, 17, 28].

In response to the growing interest in exoskeletons to enhance the outcomes of neurorehabilitation, particularly in SCI/D, a significant body of literature has been published on associated topics and therapeutic benefits such as cardiovascular function [19], gait performance and training [17, 19], spasticity and pain [18], device characteristics [29], cardiorespiratory function and fatigue [30]. Although the aforementioned evidence is based on structured rehabilitation protocols, little emphasis has been given to discussing dose and dosage parameters of the exercises used in the respective therapeutic protocols beyond feasibility, safety and the specific outcomes observed. Additionally, interventions using powered exoskeleton-based rehabilitation for gait and balance were reportedly delivered under widely variable designs [31–34]. Although dose and dosage parameters were reported by previous systematic review authors in adults with SCI/D who underwent lower limb powered exoskeleton rehabilitation for overground gait and balance, most did not discuss these training parameters. Instead, most authors acknowledged the absence of best practice recommendations in the field and endorsed the need to further understand rehabilitation designs aimed to restore or maintain locomotion with powered exoskeletons [7, 15, 21, 35].

This systematic review addresses two main questions: (1) To what extent are dose (e.g., single session duration, and total number of sessions) and dosage (e.g., frequency of sessions per week, and total duration of the intervention) of exoskeleton-based exercises reported in the literature on overground gait and balance rehabilitation for adults with SCI/D (subacute or chronic, complete or incomplete)?; and, (2) Which outcome measures are used to inform changes in gait and balance following exoskeleton-based rehabilitation in SCI/D? We hypothesized that the investigation of dose and dosage parameters of exoskeleton-based exercises reported from interventions for overground gait and balance rehabilitation interventions among individuals with SCI/D would contribute to: (1) the identification of consistent dose and dosage parameters to inform best practice recommendations related to locomotor rehabilitation strategies; and, (2) informing the development of innovative, clinically robust protocols evaluating exoskeletons for SCI/D rehabilitation; and, (3) to driving implementation of exoskeleton based training programs within tertiary SCI/D rehabilitation settings.

## Methods

This systematic review was conducted in accordance with the Preferred Reporting Items Systematic Reviews and Meta-Analyses (PRISMA) guidelines [36] and registered in the International Prospective Register of Systematic Reviews (PROSPERO) under the number CRD42022319271.

### Search strategy and data sources

The search strategy was co-developed by the authors in collaboration with a local Medical Librarian and Information Specialist (MP) using the concepts contained in the PICO framework encompassing **P**opulation, **I**ntervention, **C**omparisons, and **O**utcomes. Valid subject headings for each database were utilized as appropriate, as were free text terms pertinent to each topic or concept (e.g., Spinal Cord Injuries; Paraplegia; Quadriplegia; Exoskeleton Device; Gait; Postural Balance). The search was performed from inception to 31 March 2022 using five electronic databases: CINAHL Complete (EBSCOhost), Embase (Ovid), Emcare Nursing (Ovid), Medline ALL (Ovid; includes PubMed non-Medline records), and the Web of Science Core Collection. Each concept searched was kept as broad as possible to ensure all relevant materials were identified. The Population encompassed adults with Spinal Cord Injuries. The Intervention was the use of Exoskeletons. The Outcomes included any biomechanical and/or clinical measures related to Gait or Balance. No date or language limits were applied. The full Medline search strategy is shown in Additional file 1.

### Study selection criteria

Studies were included according to the following criteria:

1. Participants: adults regardless of sex/gender identity (≥ 16 years of age) with subacute/chronic (≥ 30 days post injury onset) complete or incomplete SCI/D of traumatic or non-traumatic etiology; and any neurological level of injury (C1-L4 ASIA Impairment Scale A-D).
2. Intervention/Exposure: overground gait and balance rehabilitation with a lower limb powered exoskeleton – an anthropomorphic device worn by the participants for orthostatic passive or active (facilitated) motor training [3].
3. Comparison: no specific rehabilitation strategy was specified for comparison.
4. Outcomes: studies which included at least 3 of 4 parameters of dose (e.g., single session duration, and total number of sessions) and dosage (e.g., frequency of sessions per week, and total duration of the intervention) of exoskeleton-based exercises; and at least one measure of gait and/or balance (e.g., Mini-Balance Evaluation Systems Test, Community Balance & Mobility Scale, ABC Scale, 6-min walk test (6MWT), 10MWT or other measure of gait speed, BBS, TUG).
5. Publication type: Experimental studies with more than five participants in randomized clinical trials, quasi-randomized clinical trials, prospective controlled trials, pre-post studies, cross-sectional, crossover and quasi-experimental studies. Studies with mixed populations (e.g., children and adults) or mixed impairments (e. g., SCI/D, stroke, multiple sclerosis), were included when outcome separation was possible. Only peer-reviewed articles were included. Reasons for exclusion included: literature reviews, qualitative studies, case series (n < 5), grey literature (i.e., letters, editorial, white papers), studies with end-effector or grounded systems, equipment design and development studies, and with gait training carried over specialized surfaces (e.g., treadmill). The inclusion and exclusion criteria are listed in the Table 1.

**Table 1:** Inclusion and exclusion criteria

Inclusion criteria	Exclusion criteria
Spinal cord injury	Reviews, letters, editorial, white papers, conference proceedings
Adults regardless sex/gender identity	Qualitative studies
16 years old or older	Grounded systems (e.g., Lokomat) and/or end-effectors
Subacute or chronic (> 30 days post injury)	Case series (n < 5)
Traumatic or nontraumatic	Mixed neurological populations if data could not be separated
Complete or incomplete	Training over a specialized surface (e.g., treadmill)
Any level of injury	Equipment design and development study
Overground gait and/or balance training with lower limb powered exoskeleton	Non overground gait
Human study	Dose and dosage not properly reported, at least 3 of 4 dose/dosage parameters
Peer-reviewed manuscripts	No gait/balance outcome measure
Including at least one outcome measures (e.g., 6MWT, 10MWT, BBS, TUG, WISCI-II, gait speed)	Acute/not possible to determine stage
Any language	Equipment design and/or development study
–	Non multi joint system

*6MWT* 6-min walk test, *10MWT* 10-m walk test, *BBS* Berg Balance Scale, *TUG* Time Up and Go, *WISCI* Walking Index for Spinal Cord Injury II

### Screening criteria and study selection

After the initial search, duplicate manuscripts were excluded, and remaining references were imported into the Covidence Systematic Review Manager (Veritas Health Innovation Ltd, Australia). Articles eligible for title and abstract screening were assessed by PN and WHS independently (a third author, KEM, was assigned to resolve eventual conflicts). Prior to working independently, an initial fidelity agreement regarding the article inclusion/exclusion process was established based on the first 10 studies with a 100% agreement between raters. If titles and abstracts did not report enough information to determine article inclusion or exclusion, the full text was screened. Following the title and abstract screening, remaining citations were independently read in full by the same two authors to verify articles met inclusion criteria. Again, disagreements were resolved by the same third author.

### Data charting and analysis

The authors created individual versions of a data extraction form. Their forms were compared and merged into a combined form used to abstract data from the included manuscripts. The data extraction form was pilot tested by two authors (PN and WHS), who independently extracted data from two of the included manuscripts. Following a comparison of the outcomes obtained, minor revisions were implemented towards a final, revised version of the abstraction form.

Data were extracted from the selected papers about authors; year of publication; institution and country of the study; participant demographics (age, number of participants, etiology and level of lesion,); dose (e.g., total number of sessions, and duration of each session, in minutes) and dosage (e.g., frequency of sessions per week, and duration of the complete intervention, in weeks); gait and balance outcomes measures (e.g., Mini-Balance Evaluation Systems Test, Community Balance & Mobility Scale, ABC Scale, 6MWT, 10MWT, BBS, TUG, gait speed). The data were synthesised by the authors and reported in tables and graphics. Narrative syntheses were applied.

In the case of articles with missing data (e.g., total duration of intervention), the corresponding author was contacted by e-mail. For some studies included, dose and dosage parameters were not explicitly stated, but could be estimated using available training parameters in the published article. For instance, sessions per week multiplied by the number of intervention weeks informed the total number of sessions; total number of sessions divided by weeks informed weekly frequency; and total number of sessions divided by sessions per week informed the duration of the intervention. For parameters indicated as best practice recommendations, only studies that reported statistically significant improvements (p < 0.05) and/or improvements equal or greater than the minimal clinically important difference (MCID) were considered. The MCID was observed for the 6MWT, 10MWT and TUG, with the following thresholds: 36 m [37] or 0.1 m/s [38], 0.13 m/s [39], and 10.8 s [40], respectively. For the cardiorespiratory outcomes, no MCID was set, and only statistically significant improvements (p < 0.05) were considered. Conversely, studies with dramatically large variability within the reported protocol (e.g., participants exposed to a different total number of sessions from 12 to 102, duration of intervention from 4 to 34 weeks) were excluded from the average calculation. As for studies with small variability within the protocol, the mean of the total range (e.g., weekly frequency from 4 to 5, was considered as 4.5; duration of each session from 60 to 90-min, was considered 75-min) were computed. Data regarding dose and dosage parameters were reported as mean and standard deviation (normal distribution) or median and interquartile range (non-normal distribution), to determine distribution the Shapiro–Wilk Test was used considering p < 0.05 as non-normal distribution.

## Results

**Fig. 1:**
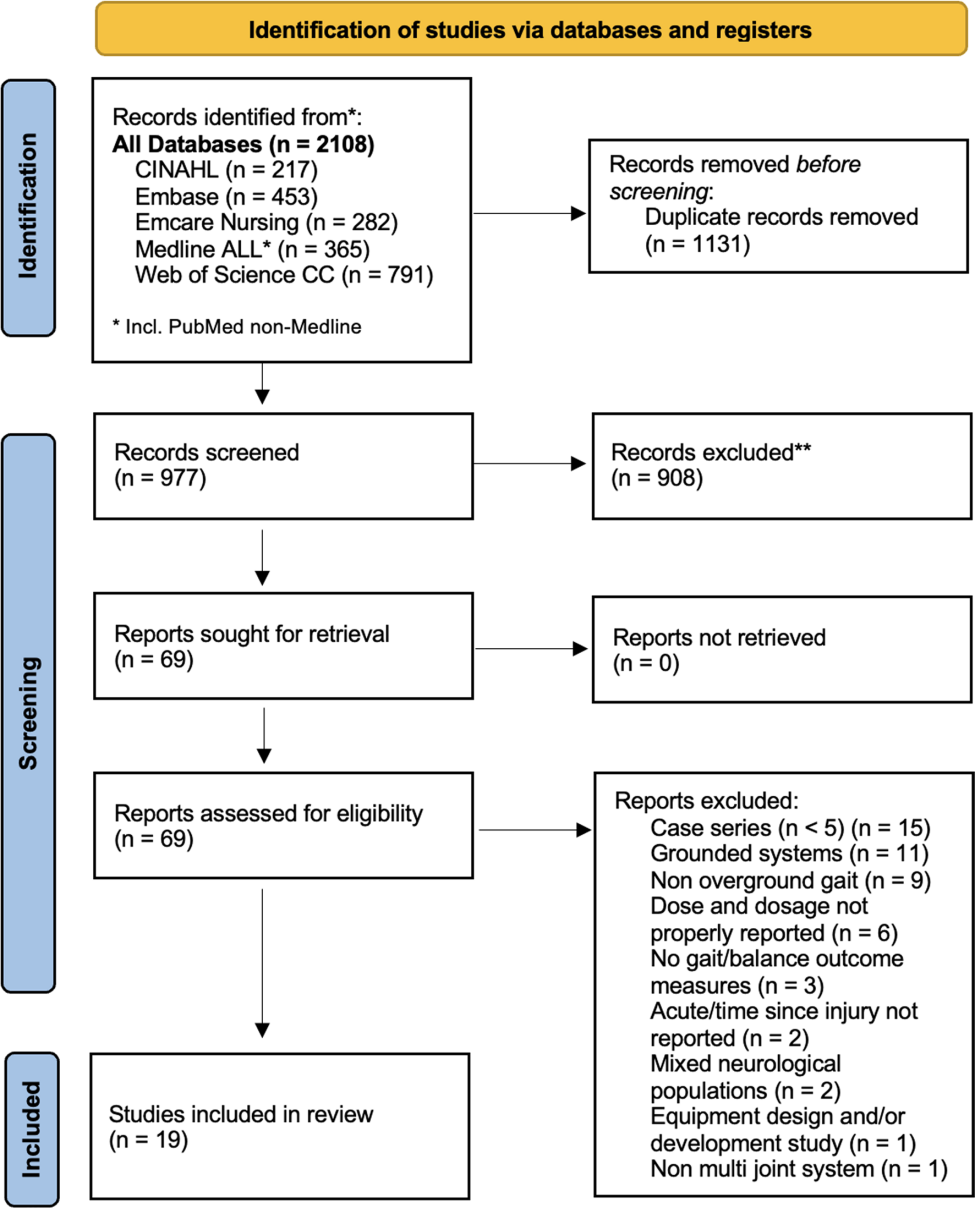
PRISMA flow diagram

**Fig. 2:**
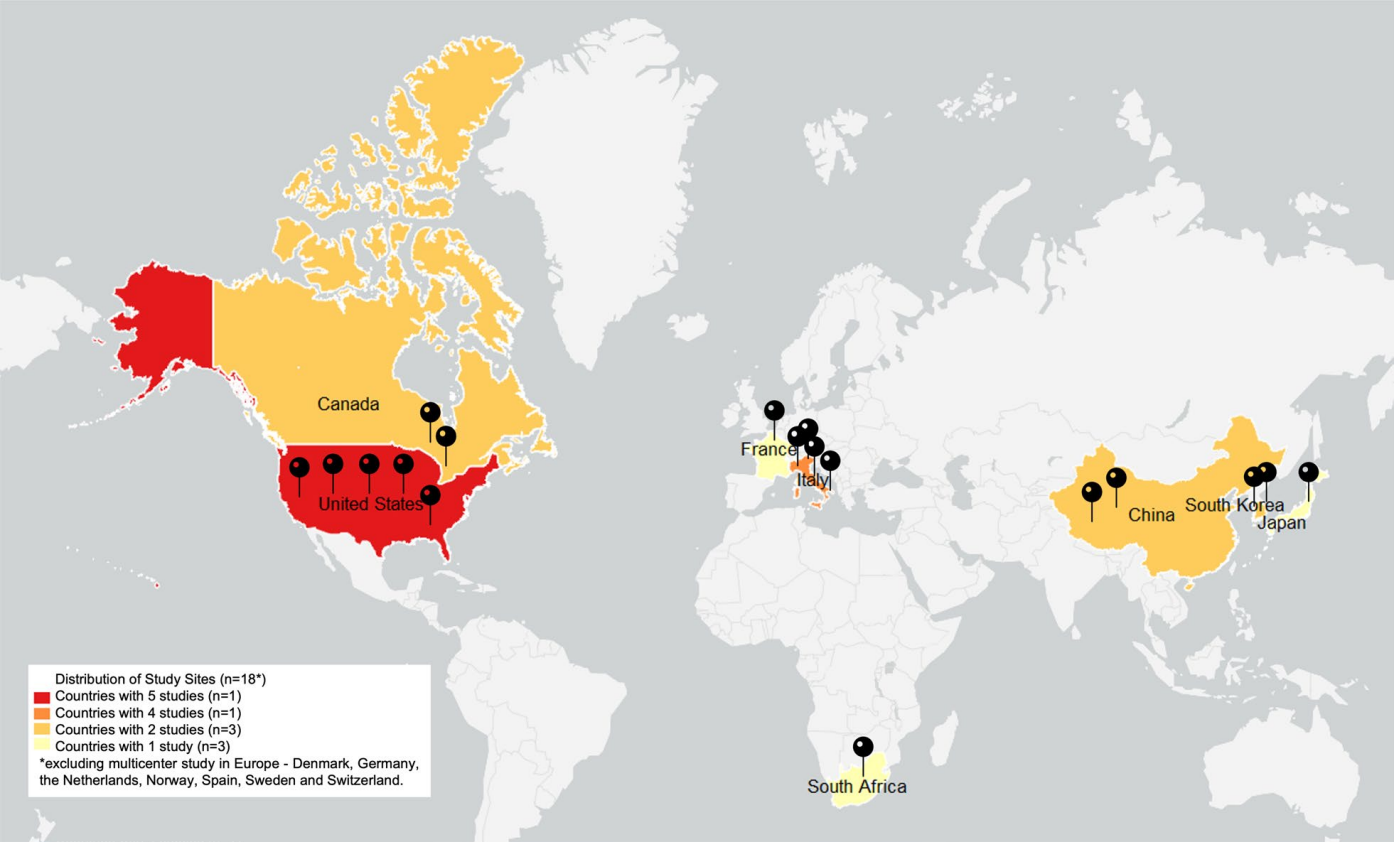
Frequency of study per country. Figure represents the country of origin of 18 of the 19 studies included because 1 study was a multicenter study across Europe

The initial electronic database search identified 2,108 references. After removing the duplicates, 977 references were screened for titles and abstracts. At full text screening, 69 articles were revised (Fig. 1). Nineteen (n = 19) full text articles were included in the review with a total of 288 participants (214 male) who underwent exoskeleton gait and/or balance training. Five (n = 5) studies had control/comparison groups treated with conventional physical therapy (n = 2) [41, 42], Lokomat gait training (n = 1) [43], BWSTT or no intervention (n = 1) [44] and BWSTT with overground gait training with functional electrical stimulation (FES) (n = 1) [45]. One (n = 1) study had a comparison group of individuals with acute SCI/D who underwent the same exoskeleton protocol [46]. As for the geographical distribution of study sites, five (n = 5) were developed in the United States [6, 33, 44, 47, 48], four (n = 4) in Italy [43, 45, 49, 50], two (n = 2) in Canada [51, 52], two (n = 2) in China [41, 53] and two (n = 2) in Korea [54, 55], one (n = 1) in France [56], one (n = 1) in Japan [46], one (n = 1) in South Africa [42], and one (n = 1) from a 7 site (Denmark, Germany, the Netherlands, Norway, Spain, Sweden and Switzerland) multicenter study in Europe [28], Fig. 2 displays the countries of origin for 18 studies, except for the multicenter study in Europe, which is the most active region investigating overground exoskeletons training for gait and balance rehabilitation among individuals with SCI/D. Six (n = 6) studies were partially or totally supported by the industry manufacturer, including equipment loan [6, 57], trial funding [28, 44, 56] and employees collaborating in manuscript production [54].

The refined dataset included articles describing participants with subacute (1 to 5 months post-injury) or chronic (> 6- or 12-months post-injury) SCI/D. Thirteen (n = 13) studies investigated chronic SCI/D (> 6 months [46, 51], > 12 months [6, 42–44, 48, 52, 54–57], stated it is chronic but did not report time since injury[50]), one study investigated subacute participants (from 1 to 11 months) [41] and five studies investigated both subacute and chronic participants [28, 45, 47, 49, 53]. The participant’s age ranged from 16 to 78 years, although one study included one participant that was 15 years old, however this paper was not included in our best practice recommendation because the authors did not find significant changes. That study, however, had a mean participant age of 41.3 years [53]. Regarding the etiology of the injury, nine (n = 9) studies included individuals with SCI/D of traumatic and non-traumatic etiology (four chronic [6, 43, 44, 51], four chronic and subacute [28, 45, 53, 57], one subacute only [41]). Five (n = 5) studies only included individuals with traumatic lesions (four chronic [42, 48, 52, 56] and one subacute and chronic [49]). One (n = 1) study focused on chronic non-traumatic participants [46]. Four (n = 4) studies did not report the etiology (three chronic [50, 54, 55], one chronic and one subacute [47]). As for the extent of injury, twelve (n = 12) studies were conducted in individuals with complete or incomplete SCI/D [28, 41, 43, 45, 47, 48, 50–54, 57], four (n = 4) studies in complete SCI/D only [6, 49, 55, 56] and two (n = 2) studies in incomplete SCI/D [42, 44]. One study did not report the extent of participant injury [46]. Relative to the level of injury, one (n = 1) study included individuals with cervical lesions [42], four (n = 4) included individuals with thoracic lesions [6, 48, 55, 56], four (n = 4) included individuals with cervical or thoracic lesions [43, 51, 52, 57], two (n = 2) included cervical, thoracic, or lumbar [44, 54], and six (n = 6) studies included thoracic or lumbar injuries [41, 45, 47, 49, 50, 53]. Two (n = 2) studies did not report the level of injury [28, 46]. A summary of participants’ characteristics and the exoskeleton device with their respective study protocols are shown in Table 2.

**Table 2:** Summary of the participants’ characteristics and exoskeleton protocols in adults with spinal cord injury

Study	Participants		Injury characteristics					Exoskeleton protocol				
	Male (n)	Female (n)	TSI (Y)	Stage	ASIA	Level	Etiology	Equipment	Total sessions	Weekly	Total intervention	Single session
Asselin et al. 2016, J Vis Exp, v. 112	10	2	1.5–19	Chronic	A-C	C8-T11	Mixed	ReWalk	12–102	3	4–34	60–90
Baunsgaard et al. 2018, Spinal Cord, v. 56	36	16	0.3 (0.2–0.4)/5.5 (2.1–10.8)^a^	Mixed	A-D	NA	Mixed	Ekso/Ekso GT	24	3	8	NA
Corbianco et al. 2021, Neurol Sci, v. 42	5	2	2–5	Chronic	A-C	C4-L2	Mixed	Ekso GT	17	2	9	60
Edwards et al. 2022, Spinal Cord, ahead of print	7	5	1.2–28.5	Chronic	C-D	C3-L4	Mixed	Ekso GT	36	3	12	45
Esquenazi et al. 2012, Am J Phys Med Rehabil, v. 91	8	4	1–24.3	Chronic	A	T3-T12	Mixed	ReWalk	24	3	8	60–90
Evans et al. 2021, Arch Phys Med Rehabil, v. 102	8	0	3–26	Chronic	C-D	C4-C7	Traumatic	Ekso GT	72	3	24	60
Gagnon et al. 2018, J Neuroeng Rehabil, v. 15	9	5	7.4 (7.8)^b^	Chronic	A-B	C6-T10	Mixed	Ekso GT	18	3	6–8	60
Guanziroli et al. 2019, Eur J Phys Rehabil Med, v. 55	11	4	0.5–15	Mixed	A	T4-L4	Traumatic	ReWalk	21.77 (4.68)^b^	3	8	60
Kerdraon et al. 2021, Spinal Cord Ser Cases, v. 7	10	2	1–18.2	Chronic	A	T5-T12	Traumatic	Atalante	12	4	3	60
Khan et al. 2019, J Neuroeng Rehabil, v. 16	8	4	1.3–24.2	Chronic	A-D	C6-T10	Traumatic	ReWalk 2.0	51.5 (6)^b^	3.7 (0.2)^b^	12	60
Knezevic et al. 2021, Arch Phys Med Rehabil, v. 102	5	0	2–14	Chronic	A-B	T1-T11	Traumatic	ReWalk	60	3	20	60
Kwon et al. 2020, Ann Rehabil Med, v. 44	8	2	1–5.4	Chronic	A	T4-T11	NA	ReWalk	20	5	4	60–90
Park et al. 2021, Sensors, v. 21	7	3	1.1–15.6	Chronic	A-C	C6-L1	NA	H-MEX	30	3	10	60
Puentes et al. 2018, Front Neurosci, v. 12	7	0	0.79–10	Chronic	NA	NA	NA	HAL	10	2	5	60
Sale et al. 2018, Eur J Phys Rehabil Med, v. 54	6	2	NA	Chronic	A-C	T1-L2	NA	Ekso GT	20	4–5	4–5	45
Stampacchia et al. 2020, Spinal Cord, v. 58	21	4	3 (1–5)^a^	Mixed	A-C	C4-L3	Mixed	Ekso	20	3	7	50–68
Tefertiller et al. 2018, Top Spinal Cord Inj Rehabil, v. 24	27	5	NA	Mixed	A-C	T4-L2	NA	Indego	24	3	8	90
Xiang et al. 2020, Spinal Cord, v. 58	19	7	0.25–14.67	Mixed	A-B	T6-L1	Mixed	AIDER	10	5	2	30
Xiang et al. 2021, J Neuroeng Rehabil, v. 18	2	7	0.17	Subacute	A/C	T4-L2	Mixed	AIDER	16	4	4	50–60

*ASIA* American Spinal Injury Association Impairment Scale, *HAL* hybrid assistive limb; n: absolute frequency, *NA* not available, *TSI* time since injury, *Y* years. ^a^: median (interquartile range); ^b^: mean (standard deviation)

### Exoskeleton training dose, dosage, and outcome measures

The 19 studies included devices from seven different exoskeleton manufacturers. Seven (n = 7) studies used Ekso devices [28, 42–45, 50, 51], six (n = 6) used ReWalk [6, 48, 49, 52, 55, 57], two (n = 2) used AIDER [41, 53], one (n = 1) used Indego [47], one (n = 1) H-MEX[54], one (n = 1) Hybrid Assistive Limb (HAL) [46] and one (n = 1) Atalante [56]. In 15 studies, the rehabilitation protocol included only exoskeleton gait and/or balance training [6, 28, 41–43, 47–54, 56, 57]. Four studies included exoskeleton training associated with overground walking without body weight supported (BWS) [44], FES cycling [45], BWS [46], or knee-ankle–foot orthosis (KAFO) gait training [55]. In respect to the dose and dosage parameters, the total number of sessions reported ranged from 10 to 102 sessions. The number of sessions per week varied from 2 to 5 sessions. The duration of the total intervention ranged from 2 to 34 weeks. The duration of each gait and balance exoskeleton gait training varied from 30 to 90-min (one paper did not report [28]). The most frequent dose and dosage parameters were: 60-min sessions [42, 43, 46, 48, 49, 51, 52, 54, 56], 3 sessions a week [6, 28, 42, 44, 45, 47–49, 51, 54, 57], over 8 to 12 weeks [6, 28, 43, 44, 47, 49, 52, 54], for a total of 20–40 sessions [6, 28, 44, 45, 47, 49, 50, 54, 55].

Overall, considering the dose and dosage parameter averages across all studies included in this review, regardless of clinically relevant change, a protocol with 60-min individual sessions, 3 times a week, for 9 weeks is suggested for a total of 27 sessions. The mean and standard deviation, or median and interquartile range for overall interventions and for protocols focused on specific therapeutic intent (e.g., functional restoration or cardiorespiratory rehabilitation) are described in Table 3. As for the total number of sessions and the duration of interventions recommended, most studies showed variability within a range of (24–36 sessions) and (8–12 weeks), respectively [6, 28, 44, 47, 49, 51, 52, 54]. Also, the duration of each session (60-min) and weekly frequency (3 times a week) were mostly consistent across the reviewed dataset, including studies with clinically relevant changes [6, 28, 42–49, 51, 52, 54, 56].

**Table 3:** Summary means and standard deviations and medians and interquartile ranges of exoskeleton protocols in adults with spinal cord injury

Parameters	Overall	Functional restoration	Cardiorespiratory
Total sessions (n)	20.9 (17–30)^a^	21.8 (18–24)^a^	35.8 (24.2)^b^
Weekly (sessions/week)	3 (3–4)^a^	3 (3–3.7)^a^	3.3 (1.0)^b^
Total intervention (weeks)	8 (4.5–10)^a^	7.5 (2.8)^b^	11.8 (8.3)^b^
Single session (minutes)	60 (57–60)^a^	60 (59–60)^a^	60 (60–60)^a^
Best practice recommendation			
Total sessions (n)	27	24	36
Weekly (sessions/week)	3	3	3
Total intervention (weeks)	9	8	12
Single session (minutes)	60	60	60

^a^: median (interquartile range); ^b^: mean (standard deviation)

**Fig. 3:**
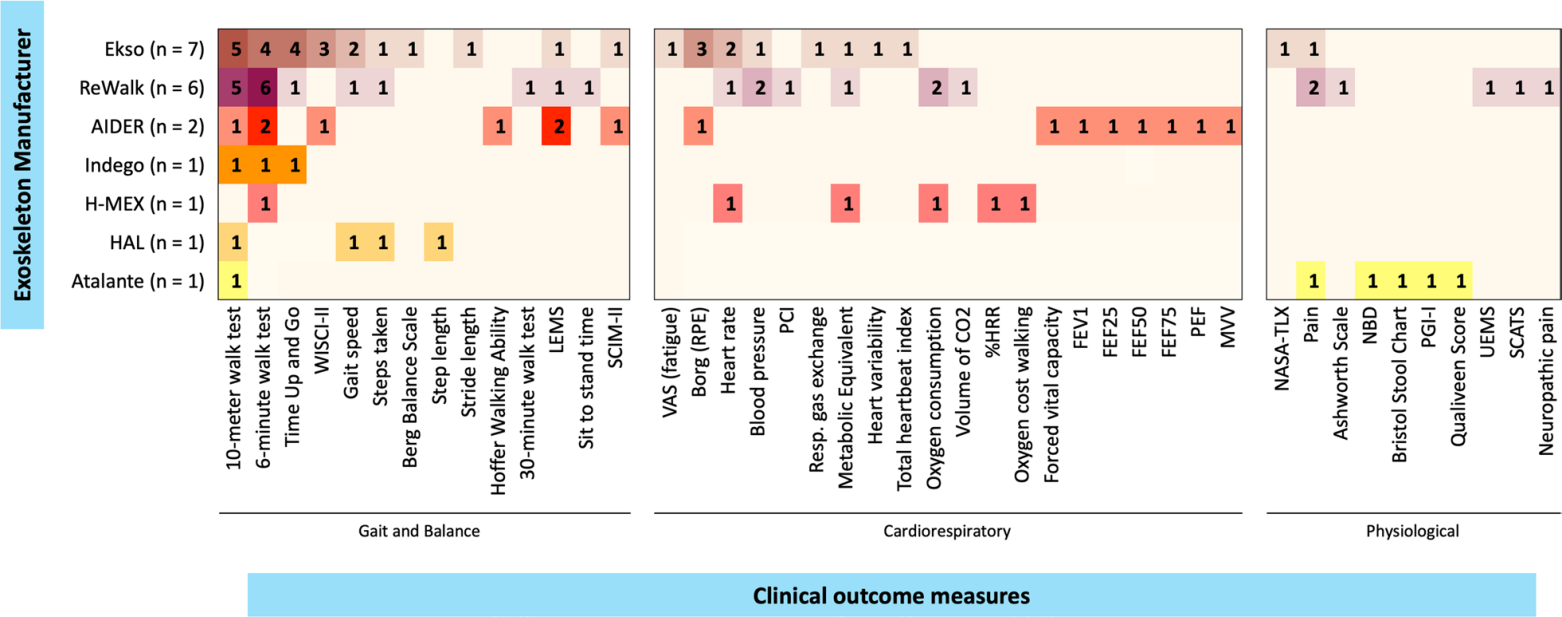
Frequency of clinical outcomes reported. Heat map presenting the frequency of clinical outcomes measures reported, per studies by manufacturer. *%HRR* percentage of heart rate reserve, *CO2* carbon dioxide, *FEF* forced expiratory flow, *FEV1* forced expiratory volume in 1 s; *LEMS* Lower Extremities Motor Score, *MVV* maximum voluntary ventilation, *NASA-TLX* NASA Task Load Index, *NBD* neurogenic bladder dysfunction, *PCI* Physiological Cost Index, *PEF* peak expiratory flow, *PGI-I* Patient Global Impression of Improvement; *Resp.* respiratory, *RPE* rating of perceived exertion, *SCATS* Spinal Cord Assessment Tool for Spastic Reflexes, *SCIM-II* Spinal Cord Independence Measure II, *UEMS* Upper Extremities Motor Score, *VAS* Visual Analogue Scale, *WISCI-II* Walking Index for Spinal Cord Injury II

The gait and balance outcome measures used include: the 6MWT [6, 41, 42, 44, 45, 47–50, 52–55, 57], 10MWT [6, 28, 44–53, 56, 57], TUG [28, 44, 45, 47, 50, 57], WISCI-II [28, 44, 45, 53], gait speed [43, 46, 50, 52], steps taken [46, 51, 52], BBS [28], step length [46], stride length [50], Hoffer Walking Ability [53], and one paper adapted the 6MWT to 30-min walk test to evaluate gait function during 30-min [55], the frequency of the gait and balance outcomes across the studies is indicated in the Fig. 3. Other non-gait related measures reported as main outcomes across different studies were categorized as either cardiorespiratory or physiologic outcomes and are listed in Fig. 3.

### Protocol therapeutic intent

The studies included in this systematic review of overground exoskeleton training dose and dosage were classified in two groups according to the inferred therapeutic intent based on the described study design which addressed: functional restoration [6, 44–47, 49–53, 56–58] or cardiorespiratory rehabilitation [41–43, 48, 54, 55]. The therapeutic intent was determined based on each study’s primary research question, aim and main outcome measures in reference to motor (gait or balance) or cardiorespiratory performance, respectively. Although in recent years changes in body composition (e.g., muscle and bone mineral density) have been increasingly associated with exoskeleton training [31, 59–61], none of the studies included in this review focused on anatomical adaptations in response to overground exoskeleton training.

#### Functional restoration

Thirteen (n = 13) studies focused on functional restoration [6, 44–47, 49–53, 56–58]. Of those, eleven reported statistically significant improvements and/or showed improvements equal or higher than the MCID for gait and/or balance outcome measures [6, 28, 44–47, 49–52, 56]. Table 4 summarizes the individual studies’ aims and main results. The functional restoration protocols ranged from 10 to 51.5 sessions, 2 to 5 sessions a week, 3 to 12 weeks of duration for 45- to 90-min. Considering the studies with significant motor improvement (n = 11), it is suggested that a protocol aimed towards functional restoration would encompass 60-min individual sessions carried 3 times a week, over 8 weeks for a total of 24 sessions (Table 3).

**Table 4:** Summary of the aim and main results of studies focused on functional restoration in adults with spinal cord injury

Study	Study goal	Results
Asselin et al. 2016, J Vis Exp, v. 112	Report the screening criteria, proper fitting, and training procedures for use a powered exoskeleton for overground walking	Participants were able to walk faster each session. There was a tendence of increase in 10MWT along the training
Baunsgaard et al. 2018, Spinal Cord, v. 56	Assess safety and feasibility during a training with exoskeletons from Ekso Bionics	Subacute improved TUG, 10MWT and BBS. Chronic improved TUG and BBS
Edwards et al. 2022, Spinal Cord, ahead of print	Demonstrate that an OG robotic exoskeleton-based gait training, can lead to an improvement in robot-independent walking speed	Gait speed increased after 12-week intervention in Ekso. 6MWT and TUG improved
Esquenazi et al. 2012, Am J Phys Med Rehabil, v. 91	Assess the safety and performance of ReWalk in enabling people with paraplegia to carry out routine ambulatory functions	Nonambulatory without wheelchair before, after all subjects walked under their own control. Spasticity seemed to be favorably impacted. Most of all reported reduction in pain
Gagnon et al. 2018, J Neuroeng Rehabil, v. 15	Investigate the feasibility and safety of a new locomotor training program with a robotic exoskeleton offered to long-term manual wheelchair users	Increases in standing time, walking time and number of steps taken and walking speed (10MWT)
Guanziroli et al. 2019, Eur J Phys Rehabil Med, v. 55	Assess walking ability of motor complete chronic SCI patients, using two different patterns of software control of wearable over-ground powered exoskeleton (ReWalk)	Participants required an average of 21.77 (standard deviation 4.68) sessions to achieve independent walking. Participants improved in 6MWT and 10MWT
Kerdraon et al. 2021, Spinal Cord Ser Cases, v. 7	Present results of the first clinical study on a newly exoskeleton that enables individuals to perform ambulatory functions without technical aids	Walking speed in 10MWT ranged from 0.06 to 0.25 m/s. All patients succeeded in standing up, sitting down, and standing up for two min
Khan et al. 2019, J Neuroeng Rehabil, v. 16	Report findings from a training program with the ReWalk, focusing on the progression in training, and the neuroplasticity induced by the training	The average number of sessions to reach walking skill were 45 sessions. Gait speed and distance covered is positively correlated to the number of sessions
Puentes et al. 2018, Front Neurosci, v. 12	Clinical scores, walking performance, and kinematics analysis was performed before and after HAL in OPLL patients after decompression surgery	HAL therapy improved the walking performance; the walking speed and stride length were increased, and the time and number of steps to cover 10 m were decreased
Sale et al. 2018, Eur J Phys Rehabil Med, v. 54	Investigate the change in gait pattern through 3D gait analysis of SCI subjects that underwent an adaptive training with an exoskeletal device	Significant improvements in TUG, 10MWT, 6MWT and 3D gait analysis. Improvements in velocity, cadence, stride length
Stampacchia et al. 2020, Spinal Cord, v. 58	Investigate whether persons affected by SCI can safely experience their walking function via RAGT and FES therapy	Participants were unable to walk at baseline. 10MWT, 6MWT, TUG and Endurance score improved significantly from the first session to the last after exoskeleton training
Tefertiller et al. 2018, Top Spinal Cord Inj Rehabil, v. 24	Assess safety and mobility outcomes utilizing the Indego powered exoskeleton in indoor and outdoor walking conditions	Improvements in 10MWT
Xiang et al. 2020, Spinal Cord, v. 58	Provide initial evidence for the effects of using a new exoskeleton as a training mobility device	6MWT increased in week 2 comparing to baseline. Individuals with motor complete had greater improvement in 6MWT and 10MWT

*10MWT* 10 m walk test; 6MWT: 6-min walk test, *BBS* Berg Balance Scale, *FES* functional electrical stimulation, *HAL* Hybrid Assistive Limb; *m* meters, *OG* overground, *OPLL* ossification of the posterior longitudinal ligament, *RAGT* robotic assisted gait training; s: seconds; *SCI* spinal cord injury, *TUG* Time Up and Go

Functional restoration interventions were shorter than cardiorespiratory interventions. They included subacute or chronic SCI/D patients, mostly with complete or incomplete thoracolumbar lesions. In this group analysis, two manuscripts did not report improvements [53, 57]. The first one [53] reported the effects of a new robotic exoskeleton based on ten 30-min sessions over 2 weeks, that is shorter than the period suggested by our recommendation based on studies with significant functional restoration gains. The second study [57] focused on describing the protocol performed in a rehabilitation research institute, including the process of participant recruitment, fitting, donning, standing, standing balance, walking, mobility training, sitting and doffing. The functional outcomes, however, were measured only after the intervention. Additionally, among the respective study participants, individuals underwent 12 to 102 sessions over 4 to 34 weeks in remarkably variable study designs.

Regarding the therapeutic content, studies on functional restoration mainly focused on sit to stand, and stand to sit transitions, standing balance and walking training for significant changes or improvements above the MCID as per functional restoration outcome measures. The frequency of training, total number of trainings, therapy content (exercise training) and studies with significant changes are shown in Table 5.A.

**Table 5:** Dose and dosage parameters, therapy content, outcome measures and effectiveness per study

A) Functional restoration							
Reference	Total sessions (n)	Single Session Duration (minutes)	Session Frequency (per week)	Therapy content		Main Outcome measures	MCID or Statistically Significant Change
				Device assistance	Exercise training		
Asselin et al. [57]	12–102	60–90	3	NA	Sit to stand training, standing upright with crutches, shifting weight laterally, mantein balance with one crutch and walking indoor and outdoor in different surfaces	6MWT, 10MWT, TUG	No
Baunsgaard et al. [58]	24	NA	3	Active, passive or active-assisted, according to the participant	Walking training with front-wheeled walker or crutches	10MWT, TUG, BBS, WISCI-II, Borg (RPE), LEMS	Yes
Edwards et al. [44]	36	45*	3	Active, passive or active-assisted, according to the participant	Walking training with exoskeleton, if minimal assistance needed overground walking without exoskeleton	10MWT, TUG, 6MWT, WISCI-II, NASA-TLX	Yes
Esquenazi et al. [6]	24	60–90*	3	NA	Sit to stand and stand to sit whitin parallel bars or with crutches and walking between parallel bars or with crutches	6MWT, 10MWT, Ashworth Scale, HR, BP, pain	Yes
Gagnon et al. [51]	18	60	3	Active mode with progression to active-assisted	Sit-stand transfers, walking and turning with forearm crutches	10MWT, steps taken	Yes
Guanziroli et al. [49]	14–33	60	3	NA	Sit-stand-sit transfers, standing balance and stepping skills and walking training coordinanting step timing and stopping	6MWT, 10MWT, sit to stand time	Yes
Kerdraon et al. [56]	12	60	4	NA	Walking training with harness without weight bearing	10MWT, NPSI, NBD, PGI-I, BSC, Qualiveen Score	Yes
Khan et al. [52]	43–66	60	3.7 (SD 0.2)	NA	Sit-stand-sit transititions, balance in standing lifting one crutch at a time or both, walking training with turining in different surfaces with crutches	10MWT, 6MWT,, steps taking, gait speed, PCI, UEMS, LEMS, pain, NPSI	Yes
Puentes et al. [46]	10	60	2	NA	Walking training in oval course at comfortable pace with harness and weight bearing, if necessary	10MWT, steps taken, gait speed, stride length	Yes
Sale et al. [50]	20	45	4–5	Active-assisted	Walking training at variable speeds	6MWT, 10MWT, TUG, stride length, VAS (fatigue), pain, Borg (RPE)	Yes
Stampacchia et al. [45]	20	50–68	3	NA	Walking training	10MWT, 6MWT, TUG, WISCI-II, SCIM-II	Yes
Tefertiller et al. [47]	24	90	3	NA	Walking training in indoor and outdoor settings	10MWT, 6MWT, TUG	Yes
Xiang et al. [53]	10	30	5	Active (maximum assistance)	Sit-stand-sit transitions and walking training with crutches	6WMT, 10MWT, WISCI-II, Hoffer Walking Ability, LEMS, SCIM-II	No
B) Cardiorespiratory focus							
Corbianco et al. [43]	17	60**	2	Active-assisted	Walking training with front-wheeled rolling walker at safety speed	Gait speed, Borg (RPE), Resp. gas exchange, HR, MET	Yes
Evans et al. [42]	72	60	3	NA	Walking training	6MWT, BP, HR, HRV	Yes
Knezevic et al. [48]	60	60	3	NA	Walking training in a hallway	6MWT, 10MWT, VO2, VCO2, BP	Yes
Kwon et al. [55]	20	60–90	5	NA	Walking training with forearm crutches	6MWT, 30MWT, MET, VO2	Yes
Park et al. [54]	30	60	3	NA	Sit-stand-sit transitions and walking training in a flat 20 m corridor with harness	6MWT, VO2, HR, MET, %HRR, oxygen cost of walking	Yes
Xiang et al. [41]	16	50–60	4	Active (maximum assistance)	Sit to stand, walking and climbing stairs reaching 40–60% of maximal heart rate	6MWT, Borg (RPE), LEMS, FEV1, FEF25, FEF50, FEF75, PEF, MVV	Yes

*%HRR* percentage of heart rate reserve; 6 min walk test; 10 m walk test; *BBS* Berg Balance Scale, *BP* blood pressure, *BSC* Bristol Stool Chart, *CO2* carbon dioxide; *FEF* forced expiratory flow, *FEV1* forced expiratory volume in 1 s, *HR* heart rate, *HRV* heart rate variability; *LEMS* Lower Extremities Motor Score, *MET* metabolic equivalent; *MVV* maximum voluntary ventilation, *NA* not available, *NASA-TLX* NASA Task Load Index, *NBD* neurogenic bladder dysfunction, *NPSI* Neuropathic Pain Symptom Inventory, *PCI* Physiological Cost Index, *PEF* peak expiratory flow; PGI-I: Patient Global Impression of Improvement; Resp.: respiratory, *RPE* rating of perceived exertion; *SCATS* Spinal Cord Assessment Tool for Spastic Reflexes, *SCIM-II* Spinal Cord Independence Measure II; *SD* standard deviation, *TUG* timed up and go, *UEMS* Upper Extremities Motor Score; *VAS* Visual Analogue Scale, *WISCI-II* Walking Index for Spinal Cord Injury II. *Donning and doffing time not included in session duration. ** Donning and doffing time included in session duration; Bold: indicates successful studies by MCID or statistically significant change

#### Cardiorespiratory rehabilitation

The six studies (n = 6) focused on cardiorespiratory rehabilitation [41–43, 48, 54, 55] showed significant improvement of cardiorespiratory function. Table 6 summarizes the cardiorespiratory studies’ aims and main results. Cardiorespiratory-centered interventions ranged from 16 to 72 sessions, 2 to 5 sessions weekly, for 4 to 24 weeks. Individual sessions lasted between 55 to 90 min. Because the six protocols yielded significant improvement in cardiorespiratory function, it is suggested that interventions to that end are likely to succeed when based on 60-min sessions carried 3 times a week for 12 weeks in a total of 36 sessions (Table 3).

**Table 6:** Summary of the aim and main results of studies focused on cardiorespiratory interventions in adults with spinal cord injury

Study	Study goal	Results
Corbianco et al. 2021, Neurol Sci, v. 42	Evaluate energy cost and psychological impact during a rehabilitation program with two different types of robotic rehabilitation systems	VO2, HR and MET were higher in Ekso. Overground walking speed increased in Ekso. Lokomat is less demanding
Evans et al. 2021, Arch Phys Med Rehabil, v. 102	Evaluate the effect of RLT and ABT on the participant’s cardiovascular indices over a 24-week intervention	One serious adverse event (tibial stress fracture). No changes in ABPI in both groups. Cardiovascular efficiency improved after exoskeleton. Increase in 6MWT after intervention
Knezevic et al. 2021, Arch Phys Med Rehabil, v. 102	Measure the cardiometabolic demands associated with EAW in persons with paraplegia	Perception of effort decreased with exoskeleton training. All participants increased 6MWT. Average VO2 increased with the progression of training
Kwon et al. 2020, Ann Rehabil Med, v. 44	Compare spatiotemporal variables and energy efficiency in an exoskeleton gait-assistive robot (ReWalk) device and KAFO in patients with paraplegia	ReWalk is significantly superior to KAFO in energy consumption in 6MWT and 30MWT. KAFO showed higher gait speed in 6MWT. ReWalk was not superior to KAFO regarding usability
Park et al. 2021, Sensors, v. 21	Investigate the exercise intensity of overground walking training with a robotic exoskeleton and to assess the changes in cardiorespiratory responses to robotic exoskeleton-assisted overground walking training	6MWT increased at mid and post-training. Oxygen cost reduced along the training
Xiang et al. 2021, J Neuroeng Rehabil, v. 18	Aimed at finding out whether the EAW trainings are different from conventional rehabilitation trainings in improving pulmonary function parameters among SCI individuals	Significant improvement in pulmonary function for exoskeleton group. No improvement in 6MWT and LEMS in exoskeleton or conventional group

*30MWT* 30-min walk test, *6MWT* 6-min walk test, *ABPI* ankle brachial pressure index, *ABT* activity-based therapy, *EAW* exoskeleton-assisted walking, *HR* heart rate, *KAFO* knee-ankle–foot orthosis; *LEMS* Lower Extremities Motor Score, *MET* metabolic equivalent of task; *RLT* robotic locomotor training, *VO2* oxygen consumption

Unexpectedly, protocols focusing on cardiorespiratory outcomes were longer in average than protocols for functional restoration. Conversely, four of the referred articles also reported significant improvements in gait and balance measures [42, 48, 54, 55], while two manuscripts reported improvements in cardiorespiratory outcomes alone [41, 43]. The latter studies were based on 16 sessions over 4 weeks [41] and 17 sessions over 9 weeks [43], indicating that shorter interventions could be enough to improve cardiorespiratory function alone, that is uncoupled from significant functional restoration. In this case, the cardiorespiratory recommendation would include 60-min sessions carried out 3 times a week for 6 weeks for a total of 18 sessions.

Regarding the therapeutic content, studies on cardiorespiratory rehabilitation mainly focused on walking training and sit to stand and/or stand to sit transitions for significant improvements in cardiorespiratory outcome measures. The frequency of training, total number of trainings, therapy content and studies with significant changes in cardiorespiratory parameters are shown in Table 5.B.

## Discussion

This review aimed to identify the dose and dosage parameters of exoskeleton-based exercises for overground gait and balance training in individuals with SCI/D. Although previous studies have discussed this topic in different neurological populations [7, 62], to the best of our knowledge, this is the first review to prioritize the investigation and discussion of dose and dosage of overground exoskeleton therapy among individuals with SCI/D – a need repeatedly acknowledged in recent literature [7, 15, 21, 35] yet widely overlooked as a primary research topic. We have summarized evidence from 19 manuscripts to determine current training parameters for specific therapeutic indications to inform best practice recommendations in exoskeleton-based SCI/D rehabilitation. Of 19 manuscripts, seventeen [6, 28, 41–52, 54–56] reported statistically significant improvements and/or gains above the MCID in the gait, balance, cardiorespiratory and/or related physiological outcomes they assessed. The evidence gathered supports the assumption that exoskeletons are a promising therapeutic tool in SCI/D, particularly for functional restoration [6, 28, 44–47, 49–52, 56] and/or cardiorespiratory improvement [41–43, 48, 54, 55].

### Protocol design

Based on strict adherence to the systematic review inclusion criteria, several manuscripts initially screened did not fully report dose (total number of sessions, and duration of the session) and dosage (frequency per week, and duration of the intervention) parameters and were excluded. Consistent with previous reviews [35, 62] on exoskeleton-based gait rehabilitation the lack of dose and dosage parameters ultimately limits the replication and generalizability of the outcomes reported. The absence of dose and dosage information also limits the translation of findings to evidence-based clinical practice, whereas the requirement for routine universal reports of dosing parameters in future studies would foster knowledge dissemination and implementation of precision rehabilitation approaches in the field. To support the development of future studies with structured information for better clinical translation, a checklist for reporting exoskeleton therapy is proposed in Table 7.

**Table 7:** Suggested checklist of essential information to include when reporting exoskeleton-based rehabilitation

Section/Topic	Item	Checklist item
Title and abstract		
	1a	Identification as an exoskeleton-based study in the title
	1b	Dose (single session duration and total number of sessions) and dosage (weekly frequency and total duration of intervention) parameters and therapeutical intent description in the abstract
Introduction		
	2a	Hypotheses regarding the exoskeleton-based rehabilitation
	2b	Specific objectives including the therapeutical intent description
Methods		
	3a	Population and characteristics’ description in detail
	3b	Participant recruitment and pre-screening process
	3c	Information on exoskeleton device (manufacturer and model)
	3d	Procedures to fit the device according to participants’ measures
	3e	Dose (single session duration and total number of sessions) and dosage (weekly frequency and total duration of intervention) parameters and therapeutical intent with the proposed protocol (e.g., functional restoration, cardiorespiratory training)
	3f	Donning and doffing time
	3 g	Pre-training participant adaptation procedures (learning to stand, walk and sit with the device)
	3 h	Assistive mode of the device used during the training, use of assistive dispositive (e.g., forearm crutches, harness, body weight support), number of therapists assisting the participant, standardize break times
	3i	Outcome measures obtained according to the therapeutical intent and when the measures were performed (e.g., pre- and post-intervention)
	3j	Adverse events registration
	3 k	Statistical methods used and additional analyses (e.g., subgroup analyses, minimal clinically important differences)
Results		
	4a	Demographic and clinical characteristic of participants
	4b	Losses and exclusions after recruitment and reasons
	4c	Results of outcome measures pre- and post-intervention
	4d	Adverse events report (e.g., pain, musculoskeletal issues, increase in spasticity, swelling in extremities, dizziness or syncope, falls, skin abrasions, numbness, fractures)

We observed considerable inconsistency of protocols for exoskeleton-based gait and balance training, with substantial variability in dose and dosage parameters used. Indeed, the protocols ranged from 10 [46] to 102 [57] sessions over 2 to 34 weeks, two [53] to five [55] times a week, with individual sessions lasting from 30 [53] to 90 min [47]. Further, the systematic review results indicated that the weekly frequency and session duration are the most consistent parameters, with most protocols reporting 3 sessions a week [6, 28, 42, 44, 45, 47–49, 51, 54, 57] at an average of 60 min per session [42, 43, 46, 48, 49, 51, 52, 54, 56]. Another important variable across studies was the device used and the exoskeleton manufacturer, with Ekso (n = 7) [28, 42–45, 50, 51] and ReWalk (n = 6) [6, 48, 49, 52, 55, 57] being the most used devices.

### Protocol effectiveness

Training effectiveness (e.g., changes in assessment values at or above the MCID) is shaped by multiple factors beyond dose and dosage, including but not limited to device parameters and the extent or intensity of training. Relative to exoskeleton-based gait and balance rehabilitation such factors include device assistance and resistance levels, different walking patterns (e.g., step and stride length, width of base of support, gait speed and step cadence) as well as exercise intensity. The latter is associated with a lack of specific consensus-based measures and definitions universally adopted by experts in the field of neurorehabilitation [63]. This is particularly true in the SCI/D populations among whom there is substantial heterogeneity in neurological impairment, and associated variability in prognosis and responsiveness to exoskeleton interventions. As a result, variability in prognosis and responsiveness are commonly observed and personalized prescriptions are provided in the absence of consensus-based terminology and practices [64, 65]. In this context, the best practice recommendations derived from this systematic review are valid given the reporting of whether the participants achieved a clinically meaningful change in function/assessment parameter based on the dose and dosage reported despite the lack of data specifying exercise intensity.

Despite protocol variability, including that of device choice, therapeutic intent, and training intensity, it is possible that the significant changes reported are associated with the repeated exposure to active standing time versus non-active sitting time [65]. However, most studies included similar functional therapeutic activities (e.g., sit to stand transitions, standing and balance training and walking training). In fact, exercise intensity in robotic rehabilitation, although not standardized, is often associated with the number of repetitions (e.g., step count), step frequency and total walking distance. The modulation of intensity on a case-by-case basis likely favored the observed performance improvements across the multiple protocol designs reported in this review. Future studies reporting the therapeutic benefits of exoskeleton therapy should include the therapeutic indication, device choice and parameters, exercise intensity, and the dose and dosage parameters as means to improve precision rehabilitation – particularly among people living with a spinal cord impairment and multimorbidity [66].

### Injury characteristics

In addition to exercise parameters, the influence of injury characteristics on exoskeleton-based SCI/D rehabilitation is very likely, yet controversial. Benson et al. [67] reported that individuals with complete injuries showed greater improvement in walking speed than incomplete injured pairs. That may be because participants with incomplete lesions were functional walkers before the beginning of their training, benefiting mostly from the ability to walk longer distances with exoskeletons as opposed to participants with complete injuries to whom exoskeletons allowed not only orthostatism, but gait initiation and speed improvements. In agreement with those findings, Xiang et al. [53] reported that individuals with higher spinal lesions and motor complete injuries showed greater improvement in gait and functional outcomes (gait speed and 6MWT) while using exoskeletons compared to people who were functional walkers with lower and or incomplete lesions. Conversely, it has been reported that adults living with lower neurological level of injury (complete versus incomplete) can achieve significantly faster walking speeds following exoskeleton training [6, 68, 69]. The explanation of these findings may be linked to the fact that people with complete SCI/D obtain more remarkable gains with training (e.g., from no standing to walking), although they still walk slower than individuals with incomplete lesions [53, 67]. Differences in gait speed is possibly associated with the remaining neural pathways in individuals with incomplete lesions, which foster better neurorecovery in response to functional restoration strategies [2]. This assumption agrees with Louie et al.’s [17] report that walking speed with exoskeletons is positively correlated with the level of spinal injury (coded from 0 (cervical) to 17 (lumbar)) and training duration. Thus, lower injuries and longer training could, favor greater locomotor gains for individuals with SCI/D. Nevertheless, Sale et al. [50] reported that exoskeleton rehabilitation is safe and feasible across a heterogeneous sample of persons with SCI/D provided it is tailored to their personal needs. Further, it is plausible that there may be additional therapeutic benefits of longitudinal training not addressed in this review.

### Exoskeleton-based therapeutic intent and physiological considerations

Upon review of the nineteen manuscripts included, consistent similarities across some of the protocols in terms of their therapeutic goals led us to classify the studies in two categories of therapeutic intent (e.g., functional restoration and cardiorespiratory rehabilitation). While the clinical purpose of individual studies seemed distinguishable enough for us to categorize them, that was not explicitly disclosed by the authors.

The current knowledge of the physiological mechanisms involved in exoskeleton-based therapies remains limited. A prior review reported that neurophysiological responses in exoskeleton recovery are linked to the exploitation of neuroplasticity, sensory stimulation, and coordination of limb and muscle activation during the training. The authors purport that functional restoration and neurorecovery are much like a relearning process where preserved sensorimotor and neural circuits are engaged to promote recovery [2]. For cardiorespiratory function, exoskeleton gait training’s rationale for the observed improvements in function associated with stimulation of the cardiorespiratory system and activation of the lower limbs is due to an increase in metabolic rate indicating this is an effective way of increasing energy expenditure with consequent improvements of cardiorespiratory fitness. Moreover, exoskeleton training contributes to the augmentation of end-systolic and end-diastolic volume, cardiac output, ventricular mass and reduces heart rate following cardiovascular conditioning [42, 54, 70].

Our findings suggest that different exercise exposures are needed to achieve MCID as per therapeutic intent in SCI/D rehabilitation, with cardiorespiratory changes demanding longer protocols compared to functional restoration. Nevertheless, we hypothesized that shorter interventions would be warranted for cardiorespiratory gains due to faster cardiovascular adaptation to structured exercises compared to neurological responses [71, 72]. This unexpected outcome may be related to two cardiorespiratory-focused manuscripts in which participants underwent longer interventions (72 [42] and 60 [48] sessions) to evaluate changes over the time (early, mid and late changes), justifying the longer experimental designs. Additionally, of the six studies [41–43, 48, 54, 55] included in cardiorespiratory rehabilitation, two [41, 43] had significant improvement in cardiorespiratory function but not in gait, which was achieved with shorter interventions, in line with our initial hypothesis. Supporting our hypothesis, Faulkner et al. [73] reported that exoskeleton gait training associated with conventional physiotherapy in 5 sessions over a single week improved cardiovascular health, by reducing the augmentation index and mean arterial pressure. Further, Evans et al. [42] reported statistically significant increases in cardiovascular efficiency as early as 6 weeks after exoskeleton gait training. Interestingly, despite protocol duration variability, the six articles focused on cardiorespiratory training reported significant improvements in cardiorespiratory health as per increased oxygen consumption, heart rate and metabolic equivalent, in addition to reduced perception of effort and oxygen cost [41–43, 48, 54, 55]. A prior systematic review reported that exoskeleton gait training elevates the energy expenditure, while allowing participants to exercise at moderate intensity, further indicating exoskeletons are beneficial for cardiorespiratory training [19].

In SCI/D, reduced lower-limb weight bearing and other health complications contribute to the loss of muscle mass and bone mineral density (BMD), specially below the level of injury [74]. This leads to an increased risk of fragility fractures, which should be accounted for when performing exoskeleton-based gait training. That is important due to previous reports of lower limb fragility fracture after exoskeleton use, mainly induced by the effect of gravity and pressure points created by the resistance of the equipment against the user’s body [75, 76]. Thus, people living with SCI/D should be advised of their fracture risk, prior to using wearable exoskeletons for increased safety, regional improvements in bone strength and BMD [59]. To prevent fragility fractures, Bass et al. [59] developed a volume and progression algorithm based on BMD thresholds. Accordingly, individuals with osteoporotic profile (T-score ≤ -2.5) should be exposed to a slow-progression program, individuals with osteopenic profile (-2.5 < T-Score < -1.0) should start with moderate-progression and individuals with preserved BMD profile (T-Score ≥ -1.0) should be enrolled in a fast-progression walking program. It is worth noting that as per the position statement 4 in the International Society for Clinical Densitometry, there is no established threshold BMD value below which weight-bearing activities are absolutely contra-indicated, and that BMD and clinical risk factors should be used together on a case-to-case basis to assess risk exposure [74]. Furthermore, people living with SCI/D are in a higher risk of developing skin abrasions and tissue injury [77]. Many studies have reported skin abrasions after the use of exoskeleton in SCI/D population [44, 53, 56, 58]. The reduction of physical activity levels, immobilization, changes in circulation and microcirculation, sensory loss, skin compression due to positioning and impaired venous return are aspects of injury that preclude individuals to lower extremity abrasions [77, 78]. Also, participants with sensory impairment are at greater risk of developing skin lesions [79], and hence warrant ongoing screening for skin integrity. That is particularly true at points of higher pressure caused the interface between the skin and the exoskeleton [44, 58, 79].

### Considerations for translation to practice

Recommendations from systematic reviews are extremely helpful at informing new research designs and guiding the translation of optimal evidence-based findings to clinical practice. However, it is also true that best practice recommendations, as identified by this review cannot always be implemented, particularly considering contextual disparities, including different countries (e.g., North America, Europe and Asia, Fig. 2), devices and therapeutic intent. Should a clinician find the implementation of the suggested best practice recommendations infeasible, reproducing the observed dose and dosage of therapy with a specific device can be limited to the shortest study with reported clinical effectiveness above the MCID for the outcome of interest (see the reduced dose and dosage but observed MCID with specific interventions on Table 5). For instance, ten 60-min sessions at a frequency of 5 sessions per week over two weeks yielded significant improvements in functional restoration [46]. Alternatively, sixteen 50–60-min sessions at a frequency of 4 times a week over four weeks yielded significant improvements in cardiorespiratory function [41]. We also suggest that patients be supported to work incrementally with healthcare providers to further implement best practice dose and dosage recommendations.

### Study limitations

This study has limitations that include the relatively scarce literature available, which did not allow us to analyze the results according to the participant’s characteristics (sex, ASIA Impairment Scale, neurologic level of injury, etc.). However, the population described in this review are similar to those described in prior reviews among individuals living with SCI/D [17, 62]. Also, it is important to state that the implementation of exoskeleton-based interventions is still limited due to the cost, availability of the equipment, equipment specifications and limitations, and the lack of highly trained staff to support exoskeleton-based therapy [80–82]. As for the limited study sample size, our search was broadened to identify manuscripts applying overground exoskeletons in SCI/D, but many of the identified references did not fully report dose and dosage – that is at least 3 parameters – and were excluded in a strategy that reduced the already restricted sample, but guaranteed data consistency. Additionally, the references included in this systematic review were classified according to their clinical intent by the review authors, which may not reflect the original authors’ intent. Furthermore, the study quality and risk of bias were not assessed as our search aimed to perform a comprehensive overview of dose and dosage in exoskeleton gait and balance training in SCI/D. Nevertheless, this systematic review is consistent with prior reports in the literature that did not report risk of bias in studies involving exoskeleton rehabilitation [7, 21, 35, 62]. The exoskeleton device donning and doffing times were inconsistently reported across the reviewed studies, with only two of them [6, 44] indicating that donning and doffing times were not part of the reported session duration and a single study [43] indicating that the session duration included donning and doffing. While we believe that some of the other sixteen studies included donning and doffing times in the session duration, we presume that most studies reported the time dedicated to standing/walking training apart from donning and doffing. Altogether, we encourage readers to implement the enclosed practice recommendations and to report device donning and doffing times, device parameters and therapeutic intensity in future reports. We also encourage clinicians and investigators to describe barriers and facilitators to implementation of best practices in different contexts.

## Conclusions

In summary, this systematic review advances the understanding of overground exoskeleton-based gait and balance training in SCI/D and its role in facilitating functional recovery and or cardiorespiratory fitness. The review results provide evidence-based clinical practice recommendations, which are tailored to the therapeutic intent of the intervention. However, problems with inconsistent reporting of exoskeleton training dose and dosage and the heterogeneity of study designs among adults with SCI/D preclude fulsome dissemination of data and are acknowledged as important limitations. To advance the field of exoskeleton rehabilitation in SCI/D and increase research quality, there is an urgent need to standardize clinical practice recommendations and guidelines through well-structured studies with clear indications of their therapeutic intent. Finally, we highlight the need for multicentre studies, which could validate the therapeutic effectiveness of specific dose and dosage parameters for optimal gait and balance rehabilitation among adults with SCI/D based on poling of data from multiple sites and contexts.

### Supplementary Information

**Additional file 1:** Medline Search Strategy. Contain the Medline Search Strategy.

## Data Availability

The datasets used and/or analysed during the current study are available from the corresponding author on reasonable request.

## Abbreviations

10MWT: 10-Meter Walk Test
6MWT: 6-Minute walk test
BBS: Berg Balance Scale
BMD: Bone mineral density
BWS: Body weight supported
BWSTT: Body weight supported treadmill training
FES: Functional electrical stimulation
HAL: Hybrid Assistive Limb
KAFO: Knee-ankle–foot orthosis
MCID: Minimal clinically important difference
PRISMA: Preferred Reporting Items Systematic Reviews and Meta-Analyses
PROSPERO: International Prospective Register of Systematic Reviews
SCI/D: Spinal cord injury/disease
TUG: Time Up and Go
WISCI-II: Walking Index SCI II
