# Supplementary material for: Exoskeleton-based exercises for overground gait and balance rehabilitation in spinal cord injury: a systematic review of dose and dosage parameters
Source: J Neuroeng Rehabil. 2024 May 5;21:73. doi: 10.1186/s12984-024-01365-2 (PMC11070073; doi:10.1186/s12984-024-01365-2)
Supplement: Supplementary file 1 — Additional file 1: Medline Search Strategy. Contain the Medline Search Strategy. [file 12984_2024_1365_MOESM1_ESM.pdf]

Addicional File 1. Medline Search Strategy

Search Strategy: Ovid MEDLINE(R) ALL <1946 to March 31, 2022>

----

1 [Population: Spinal Cord Injured]
2 exp Spinal Cord Injuries/ (52932)
3 exp Paraplegia/ (13347)
4 exp Quadriplegia/ (8287)
5 (spinal cord adj3 (injur* or contusion* or trauma* or transection* or lacerat* or compression* or lesion*)).mp,kw. (74792)
6 (paraplegi* or quadrip* or tetrap* or SCI).mp,kw. (105893)
7 (myelopath* adj2 (traumatic or post-traumatic or post traumatic or compressive)).mp,kw. (814)
8 or/2-7 (154191)
9 [Intervention: Exoskeletons]
10 Exoskeleton Device/ (1130)
11 exoskelet*.mp,kw. (4250)
12 (robot* adj3 (device* or walk* or gait* or rehab* or lower-limb* or "lower limb" or lower-extremit* or "lower extremit*" or locomotor*)).mp,kw. (4664)
13 or/10-12 (8155)
14 [Outcomes: Gait or Balance Related]
15 exp Gait/ (33967)
16 exp Postural Balance/ (26594)
17 (gait* or balanc*).mp,kw. (442216)
18 or/15-17 (442710)
19 8 and 13 and 18 (365)

****
